# Phytoestrogen (+)-pinoresinol exerts antitumor activity in breast cancer cells with different oestrogen receptor statuses

**DOI:** 10.1186/s12906-016-1233-7

**Published:** 2016-09-07

**Authors:** Alicia López-Biedma, Cristina Sánchez-Quesada, Gabriel Beltrán, Miguel Delgado-Rodríguez, José J. Gaforio

**Affiliations:** 1 Center for Advanced Studies in Olive Grove and Olive Oils. Agrifood Campus of International Excellence (ceiA3), University of Jaén, Campus Las Lagunillas s/n, 23071 Jaén, Spain; 2 Instituto Andaluz de Investigación y Formación Agraria, Pesquera y de la Producción Ecológica (IFAPA), Centro “Venta del Llano”, 23620 Mengíbar, Jaén Spain; 3 CIBER-ESP, Ministry of Health, Madrid, Spain

**Keywords:** Cytotoxic activity, Antioxidant, Virgin olive oil, Polyphenols, Chemopreventive

## Abstract

**Background:**

Consumption of virgin olive oil (VOO) has been associated with a low breast cancer incidence. Pinoresinol is a phytoestrogen that is typically found in VOO. Considering the role of oestrogen in breast cancer development and progression, we investigated the potential antitumor activity of pinoresinol in breast cancer cells.

**Methods:**

To address this question, we treated MDA-MB-231 (oestrogen receptor [ER] negative) and MCF7 (ER+) human breast tumour cells and MCF10A human mammary epithelial cells (ER-) with different concentrations of pinoresinol. The cytotoxic activity, cell proliferation, cell cycle profile, apoptosis induction, reactive oxygen species production and DNA damage were assessed.

**Results:**

Pinoresinol showed cytotoxic, anti-proliferative and pro-oxidant activity in human breast tumour cells, independent of their oestrogen receptor status. In addition, pinoresinol exerted antioxidant activity and prevented DNA damage associated with oxidative stress in human mammary epithelial cells.

**Conclusions:**

Overall, the results suggest that pinoresinol may have antitumor activity in human breast cancer cells independently of oestrogen receptor status. Furthermore, the results show that the pinoresinol has the typical characteristics of a chemopreventive compound.

**Electronic supplementary material:**

The online version of this article (doi:10.1186/s12906-016-1233-7) contains supplementary material, which is available to authorized users.

## Background

Growing scientific evidence suggests that the intake of virgin olive oil (VOO), which is the main source of fat in Mediterranean diets, correlates with a low incidence of breast cancer [1]. Among the minor compounds present in VOO that possess different health properties [2–6], we find polyphenols to be a very interesting group because of their biological benefits. It has been reported that polyphenols prevent the development and progression of pathological conditions, such as cancer, neurological and cardio-vascular diseases, diabetes, aging, and so on [7].

**Fig. 1:**
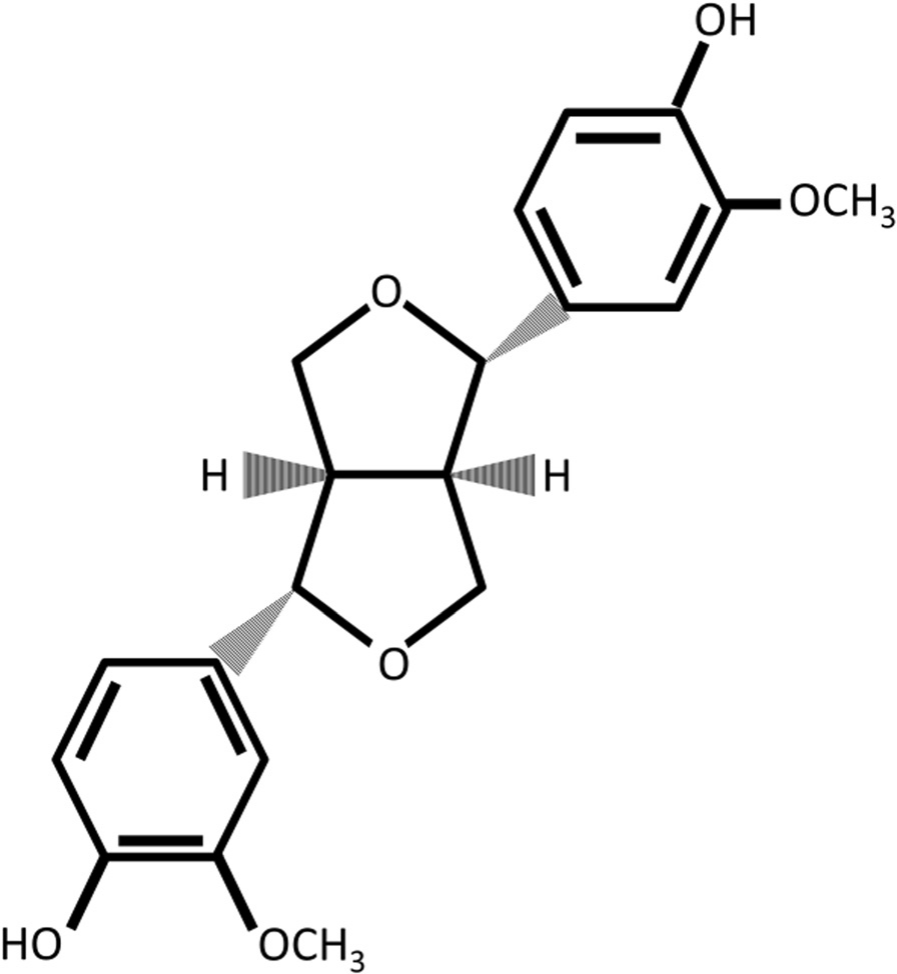
Chemical structure of (+)-pinoresinol

One of the most abundant phenolic compounds in VOOs, behind tyrosol and hydroxytyrosol, is (+)-pinoresinol (PINO) [8] (Fig. 1). Its presence in VOOs depend on the variety of the cultivar, in amounts of 0.07 ± 0.003 mg/kg in Arbequina variety, about 0.90 ± 0.78 in Picual variety [9]. Brenes et al. [10] reported that Spanish olive oil contains a range of 20 to 45 mg/kg PINO. Several health properties have been attributed to PINO, including antifungal [11], anti-inflammatory [12, 13], hypoglycaemic [14] or chemopreventive biological activities [15, 16].

PINO has a chemical structure that is similar to that of oestrogen (i.e., it is a phytoestrogen). Oestrogen is essential for the growth and development of mammary glands and has been linked with the development and progression of breast cancer due to enhanced binding and activation of the oestrogen receptor α (ERα) [17]. For example, the phytoestrogen tamoxifen acts as an oestrogen antagonist in breast tissue and has been shown to slow breast cancer cell proliferation and has been used in clinical practice for breast cancer patients [18].

Interestingly, ERβ has also been shown to mediate estrogenic action. The specific role of this receptor in human breast cancer remains elusive; however, in contrast to ERα, ERβ has been linked with anti-proliferative and pro-apoptotic activities. In fact, the expression of ERβ is lower in human breast cancer cells compared to normal breast cells, supporting its potential tumour-suppressive role [19].

Surprisingly, very few studies have noted the role of PINO as a potential agonist or antagonist of oestrogen and the chemopreventive repercussions that PINO treatment may have on hormone-related breast cancer [20].

The chemical antioxidant activity of PINO also remains unclear. A few studies using DPPH and ABTS assays have shown different antioxidant functions of PINO [21-23]. However, the Oxygen Radical Absorbance Capacity method (ORAC) has not been used in past studies, despite being considered one of the most biologically relevant assays [24].

Furthermore, the little research that has been done surrounding the effects of this compound on breast cancer cells remains inconclusive. Chin et al. [21] described a lack of cytotoxic effects and a cytoprotective effect of PINO on MCF7 cells stressed by H_2_O_2_ [25]. Other authors have reported anticancer effects of PINO by suppressing the expression of the lipogenic enzyme FASN in HER-2 overexpressing MCF7 cells [26]. Recently, Sepporta et al. [27] observed that PINO inhibited the growth of MDA-MB-231 cells, but not of MCF7 cells. Importantly, no previous study has examined the effects of PINO on a normal human breast cell line, which would address whether PINO plays a protective role against cancer development.

Therefore, the aim of the present study was to examine whether PINO exerts chemopreventive and/or antitumor activity in breast cancer, specifically because this compound is found in VOO and its consumption has been related with a minor incidence of breast cancer.

Therefore, to determine whether this compound may contribute, at least in part, to the health benefits attributed to VOO on breast cancer incidence and mortality, we studied the effects of PINO on breast cells with different receptor expression patterns. For this purpose, we used the following human mammary cells: highly invasive MDA-MB-231 (oestrogen receptor [ER] and progesterone receptor [PR] negative) breast tumour cells, the minimally invasive MCF7 (ER and PR positive) breast tumour cells and MCF10A human mammary epithelial cells (ER and PR negative).

## Methods

### Chemicals and material

The following were purchased from Gibco® Life Technologies Ltd (Paisley, UK): HuMEC Ready Medium (1X), TrypLE™ Express Enzyme (1X) and Minimum Essential Medium (MEM). Foetal bovine serum (FBS) was obtained from PAA Laboratories GmbH (Pasching, Austria). Ethanol 96 % v/v and potassium peroxodisulfate (K_2_S_2_O_8_) (CAS 7727-21-1) were purchased from Panreac Química S.L.U. (Barcelona, Spain). The CellTiter-Blue® Cell Viability Assay was acquired from Promega Corporation (Madison, WI, USA). Round bottom culture plates and cell culture flasks were purchased from Nunc A/S (Roskilde, Denmark). Flat bottom culture plates were from CytoOne (Hamburg, Germany). Fluorescein (FL) (CAS 2321-07-5) was obtained from Life Technologies (Carlsbad, CA, USA). The following were obtained from Sigma-Aldrich Co. (St. Louis, MO, USA): (+)-Pinoresinol (CAS 487-36-5) purity ≥95 %; PBS; (S)-(+)-camptothecin (CPT) (CAS 7689-03-4) purity ≥90 %; 2′,7′-dichlorofluorescin diacetate (DCFH-DA) (CAS 4091-99-0) purity ≥97 %; Sodium pyruvate solution (CAS 113-24-6); MEM Non-essential Amino Acid Solution (NEAA); HEPES buffer solution (CAS 7365-45-9); 2,2-Diphenyl-1-picrylhydrazyl (DPPH) (CAS 1898-66-4); 2,2′-Azino-bis(3-ethylbenzothiazoline-6-sulfonic acid) diammonium salt (ABTS) (CAS 30931-67-0) purity ≥98 %; (±)-α-Tocopherol (Vitamin E) (CAS 10191-41-0) purity ≥96 %; (±)-6-Hydroxy-2,5,7,8-tetramethylchromane-2-carboxylic acid (Trolox™) (CAS 53188-07-1) purity 97 % and 2,2′-Azobis (2-methylpropionamidine) dihydrochloride (AAPH) (CAS 2997-92-4) purity 97 %. PBS (1X, Dulbecco’s) and DMSO (CAS 67-68-5) were obtained from AppliChem GmbH (Darmstadt, Germany). The PI/RNase Staining Buffer kit, Annexin V-FITC kit and Comet Assay kit (CAS 50-07-7) were purchased, respectively, from BD Biosciences, Pharmingen (San Diego, CA), Miltenyi Biotec (Bergisch Gladbach, Germany) and Trevigen, Inc. (Gaithersburg, MD, USA). Non-tumorigenic human breast epithelial cells (MCF10A), minimally invasive human breast cancer cells (MCF7) and highly invasive human breast cancer cells (MDA-MB-231) were obtained from American Type Culture Collection (ATCC, Manassas, VA, USA).

### ABTS radical scavenging test

The ABTS radical scavenging activity was measured as previously reported [28]. ABTS radical cations (ABTS^●+^) were produced by reacting 7 mM ABTS with 2.45 mM K_2_S_2_O_8_ (final concentration) for 16 h in the dark at room temperature. The radical obtained was diluted in ultrapure water until the absorbance at 734 nm was between 0.7 and 1. Ethanol solutions of Trolox™ (antioxidant standard) or pinoresinol (PINO) were diluted in ultrapure water to reach concentrations between 50 and 800 μM and 0.00001 and 1000 μM, respectively. Twenty microliters of each concentration of Trolox™, PINO, ultrapure water (blank) or ethanol control (10 %) were added into a flat bottom 96-well plate. The reaction was initiated by the addition of 50 μL of ABTS^●+^, and the absorbance at 734 nm was immediately measured every 5 min over 2 h at 30°C with a TECAN GENios Plus microplate reader (Tecan Group Ltd., Switzerland). All of the reactions were performed in triplicate, in three independent experiments. The percentage of the radical scavenging activity (% RSA) was calculated according to the following formula:

1 $$ \%\ \mathrm{R}\mathrm{S}\mathrm{A} = 100\left({\mathrm{A}}_{\mathrm{C}(0)}\hbox{--}\ {\mathrm{A}}_{\mathrm{A}\left(\mathrm{t}\right)}\right)/{\mathrm{A}}_{\mathrm{C}(0)} $$

where A_C(0)_ is the absorbance of the blank at t = 0 and A_A(t)_ is the absorbance of the compound/standard at t = 60.

### Radical scavenging activity by the DPPH assay

Estimation of the antioxidant capacity against the radical DPPH was carried out according to Brand-William et al., [29] with some modifications. An ethanolic solution of DPPH 100 μM (final concentration) was mixed in 96-well plates with ethanolic solutions of PINO or α-tocopherol (antioxidant standard) at 0.03, 0.06, 0.13, 0.25, 0.5, 1 and 2 mole ratios (moles of antioxidant/moles of DPPH). DPPH samples without antioxidants were also measured as blank controls. The absorbance at 520 nm was read every 5 min over 2 h with a TECAN GENios Plus microplate reader. Measurements were performed at least in triplicate in three separate experiments. The radical scavenging activity (% RSA) was calculated as described in Eq. (1) (t = 60).

### ORAC_FL_ assay

The Oxygen Radical Absorbance Capacity (ORAC) of PINO was assayed as described in Prior et al. [30]. This method measures the oxidative degradation of fluorescein induced by the thermal decomposition of the AAPH azo-compound. In brief, fluorescein (48 nM) was added to each well of a round bottom 96-well plate that was previously tempered at 37°C. Then, PINO (from 0.001 to 1000 μM), Trolox^TM^ (standard, from 12.5 to 100 μM) or PBS (blank) with a final volume of 1 % DMSO (v/v) was added to the wells. After incubating for 15 min at 37 °C, AAPH was added to the wells. Fluorescence readings (Ex. λ_485_/Em. λ_520_ nm) were taken every 5 min at 37 °C for 160 min with a TECAN GENios Plus microplate reader. The final results were calculated based on the difference in the area under the fluorescence decay curve (AUC) between the blank and each sample. The AUC formula was determined as follows:

2 $$ AUC = 1 + {f}_1/{f}_0+{f}_2/{f}_0+{f}_3/{f}_0 + \dots +{f}_n/{f}_0 $$

where *f*_0_ is the initial fluorescence at cycle 0 and *f*_n_ is the fluorescence reading at cycle *n*.

The results were expressed as micromolar Trolox^TM^ equivalents (TE), which were calculated using the line equation from the standard curve:

3 $$ TE = \left(Y\ \hbox{--}\ b\right)/m $$

where *Y* is the net AUC (AUC_sample_ – AUC_control_), *b* is the *Y*-intercept and *m* is the slope.

### Cell culture and treatments

Human MCF10A (ERα and PR negative) breast epithelial cells were grown in HuMEC Ready Medium. Human MCF7 (ERα and PR positive) and MDA-MB-231 (ERα and PR negative) breast cancer cells were grown in MEM supplemented with 10 % FBS, 1 % Hepes buffer, 1 % NEAA and 1 % Sodium Pyruvate. The cells were cultivated as monolayer cultures in a humidified atmosphere with 5 % CO_2_ at 37°C and subcultured using TryPLE Express. Cells growing between 90 and 95 % of confluence were used for all experiments. The cells were treated for 24 h with 0.001, 0.01, 0.1, 1, 10 and 100 μM of PINO that was previously dissolved in DMSO (stock concentration 50 mM).

### Cytotoxicity assay

The effects of PINO on cell viability were determined by the CellTiter-Blue® Cell Viability Assay according to the manufacturer’s protocol with some modifications. A total of 5x10^3^ cells/well (for MDA-MB-231 and MCF7) or 2.5x10^3^ cells/well (for MCF10A) were seeded onto a 96-well plate. After 24 h to allow for cell attachment, the cells were treated with PINO or DMSO (as vehicle control) for another 24 h. CellTiter-Blue® was then added, and the plates were incubated for 3 h in darkness at 5 % CO_2_ and 37°C. Finally, fluorescence was read with a TECAN GENios Plus microplate reader (Ex. λ_485_/Em. λ_595_ nm) and viability was calculated using the formula:

4 $$ \%\  viable\  cells = \left[\left({A}_{treated\  cells}\right)/{A}_{control}\right]\ x\ 100 $$

where *A* corresponds to the relative fluorescence units of each sample. All of the measurements were performed in triplicate and each experiment was repeated at least three independent times.

### Cell proliferation assay

In all of the cell proliferation experiments performed, the cells were seeded cells onto 96-well plates and allowed to attach before adding PINO or DMSO as the vehicle control. After 24 h of treatments, the medium was replaced by fresh medium and the plates were incubated for another 24 h. Then, CellTiter-Blue® was added, and fluorescence was read after 3 h of incubation with a TECAN GENios Plus microplate reader (Ex. λ_485_/Em. λ_595_ nm). The measurements were repeated at 48, 72 and 96 h. The percentage of viable cells was calculated as defined in Eq. (4).

### Cell cycle analysis

A total of 1 x 10^5^ cells/mL (for MDA-MB-231 and MCF7 cells) or 5 x 10^4^ cells/mL (for MCF10A cells) were seeded and allowed to attach for 24 h before treating with PINO for another 24 h. The cells were then fixed in cold 70 % ethanol, stored at −20°C for at least 24 h and labelled with a PI/RNase Staining Buffer kit. Cell cycle assessment was conducted by flow cytometry in an EPICS XL-MLC flow cytometer (Beckman Coulter, Spain), and the results were analysed using the FlowJo program (v5.7.2). Each experiment was repeated three independent times.

### Apoptosis analysis

MDA-MB-231 (1 x 10^5^ cells/mL), MCF7 (1 x 10^5^ cells/mL) or MCF10A (5 x 10^4^ cells/mL) cells were seeded, allowed to attach and treated for 24 h with PINO. The cells and supernatants were collected and labelled with Annexin V-FITC kit according to the manufacturer’s suggestions. As a positive control, the cells were incubated with 1 μM camptothecin (CPT). Apoptosis analysis was carried out using an EPICS XL-MLC flow cytometer, and the results were analysed using the FlowJo program. Each experiment was repeated three independent times.

### Detection of reactive oxygen species

Detection of intracellular Reactive Oxygen Species (ROS) was performed using the probe 2’, 7’-dichlorofluorescin diacetate (DCFH-DA) as previously reported by our group [31]. In brief, MCF10A (5.5x10^3^ cells/well), MDA-MB-231 or MCF7 cells (7x10^3^ cells/well) were seeded onto 96-well plates, allowed to attach for 24 h and then treated with PINO for an additional 24 h. After the addition of DCFH-DA (100 μM), the plates were incubated for 30 min at 37 °C and 5 % CO_2_. Fluorescence was then read for 30 min (Ex. λ_485_/Em. λ_535_) with a TECAN GENios Plus microplate reader.

It is well known that the addition of H_2_O_2_ increases stress in culture cells [32]. To test whether PINO had a protective role against induced oxidative stress, the assay was also performed after the addition of H_2_O_2_ (400 μM) 30 min before quantification.

Both experimental conditions were assayed three independent times, and each measurement was performed in quadruplicate. In all cases, iron free media (MEM or HuMEC) were used.

The intracellular ROS level percentage was calculated as follows:

5 $$ F = \left[\left(F{t}_{30}\hbox{--} F{t}_0\right)/F{t}_0\right]\ x\ 100 $$

where *Ft*_0_ is the fluorescence at *t* = 0 min and *Ft*_30_ the fluorescence at *t* = 30 min.

### Alkaline single-cell gel electrophoresis (Comet assay)

To estimate the state and wholeness of DNA, 5x10^4^ cells/well (for MCF10A cells) or 1x10^5^ cells/well (for MCF7 and MDA-MB-231 cells) were allowed to attach to a 12-well plate and treated with increasing PINO concentrations for 24 h. The cells were then detached and centrifuged twice in PBS. To evaluate whether PINO had the ability to protect against oxidative DNA damage, cells were also exposed to H_2_O_2_. The comet assay was carried out according to Warleta et al. [3]. Analysis of the DNA strands was performed by examining twenty-five random cell images per sample in a Zeiss Axioplan 2 epifluorescence microscope (Carl Zeiss; Jena, Germany) equipped with Luca EMCCD camera (Andor Technology, Belfast, UK) and using the Komet 5.5 software package (Kinetic Imaging Ltd., Liverpool, UK). DNA damage was calculated by determining the relative fluorescence between the head and tail using the olive tail moment (Olive_TM), which was defined as:

6 $$ Olive\_TM = \left[\left( tail\ (mean)\ \hbox{--}\ head\ (mean)\right)\ x\  tail\ \left(\%\ DNA\right)\right]/100 $$

### Statistical analysis

Statistical analyses were performed using one-way analysis of variance (ANOVA) followed by Fisher’s LSD test with the STATGRAPHICS Centurion XVI software (Statpoint Technologies, Inc. Warrenton, VA, USA). The values of *p* < 0.05 were considered significant. The data are represented as the mean of at least three independent experiments ± SEM and are expressed relative to the untreated controls.

## Results

### ABTS radical scavenging test

**Fig. 2:**
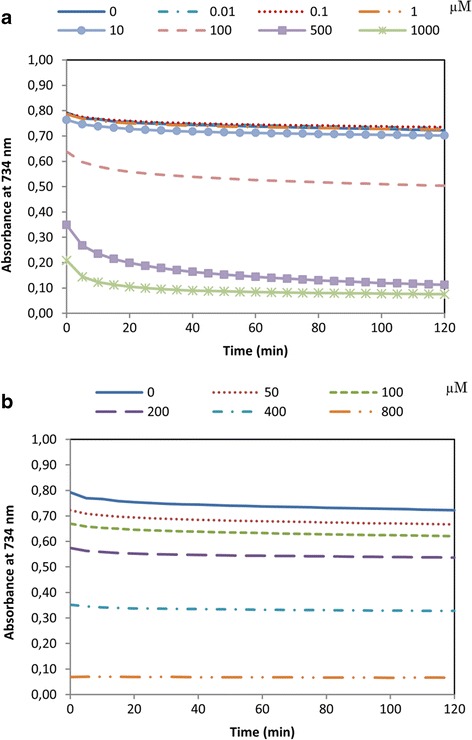
ABTS radical scavenging assay. ABTS^●+^ radical scavenging activity of (+)-pinoresinol **a** or Trolox^TM^ **b** at concentrations between 0.01and 1000 μM and 50 and 800 μM, respectively. Data are expressed as the absorbance of ABTS^●+^, which was read at 734 nm for 2 h. Measurements were performed in triplicate in three independent experiments

The radical cation ABTS^●+^ was diminished by pinoresinol (PINO) above 10 μM (Fig. 2), and the studied range was from 0.00001 to 1000 μM. Concentrations lower than 10 μM did not shown antioxidant capacity (data not shown). The antioxidant effects of PINO were higher than the antioxidant standard, as the 50 % of Radical Scavenging Activity (RSA) occurred at 380 μM for Trolox™ and at 274 μM for PINO.

### Radical scavenging activity by the DPPH assay

**Fig. 3:**
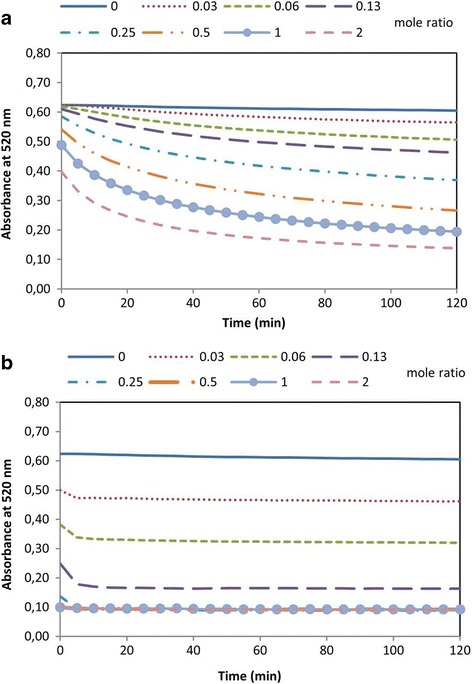
DPPH radical scavenging assay. Antioxidant activity of (+)-pinoresinol **a** against DPPH radicals at 0.03, 0.06, 0.13, 0.25, 0.5, 1 and 2 mole ratios (mol antioxidant/mol DPPH). α-tocopherol **b** was used as the antioxidant control at the same ratios. Data represents the absorbance of DPPH radicals read at 520 nm for 2 h. Each experiment was performed in triplicate and repeated three times

As depicted in Fig. 3, PINO exhibited antioxidant activity against the DPPH radical in a dose dependent manner. The RSA of PINO was determined to be 50 % at 0.69 mol ratio (69 μM), while the RSA of the antioxidant control α-tocopherol was 50 % at 0.11 mol ratio (11 μM).

### ORAC_FL_ assay

The peroxyl radical scavenging activity of PINO, as measured by ORAC_FL_, showed a protective effect against AAPH-induced peroxyl radical activity. PINO exerted a higher protection than Trolox™. The micromolar Trolox^TM^ equivalents (TE) values were 39.95, 64.93, 114.89 and 214.81 for 12.5, 25, 50 and 100 μM of PINO.

### Cytotoxicity assay

**Fig. 4:**
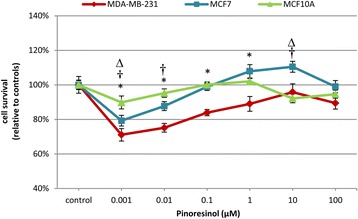
Cytotoxicity assay. Cell survival measured by CellTiter-Blue® after 24 h of (+)-pinoresinol treatment on MDA-MB-231, MCF7 and MCF10A cells. Data are represented as the treatment average (±SEM) with respect to the control, which was considered as 100 %, for three independent assays carried out in triplicate. * MDA-MB-231, † MCF7 and ∆ MCF10A indicate statistically significant differences at *p* < 0.05

To assess the potential cytotoxic effects of PINO, MDA-MB-231, MCF7 and MCF10A cells were treated with concentrations of PINO ranging from 0.001 to 100 μM for 24 h. Surprisingly, PINO treatment was shown to promote a widespread cytotoxic effect at low concentrations in MCF7 cells and at all of the concentrations tested in MDA-MB-231 cells, although statistically significant changes were only observed from 0.001 to 1 μM (Fig. 4). Importantly, the percentage of non-tumorigenic human mammary epithelial cells death following treatment with 0.001 μM PINO was much lower (10 %) than in breast cancer cells (29 % for MDA-MB-231 and 20 % for MCF7 cells). In addition, a 10 μM PINO dose was shown to inhibit proliferation in MCF7 cells but did not induce significant cytotoxicity in MCF10A. Interestingly, a statistically significant cytotoxic effect was observed following 0.01 μM PINO treatment in both types of human breast tumour cells tested, but not in human mammary epithelial cells.

### Cell proliferation

**Fig. 5:**
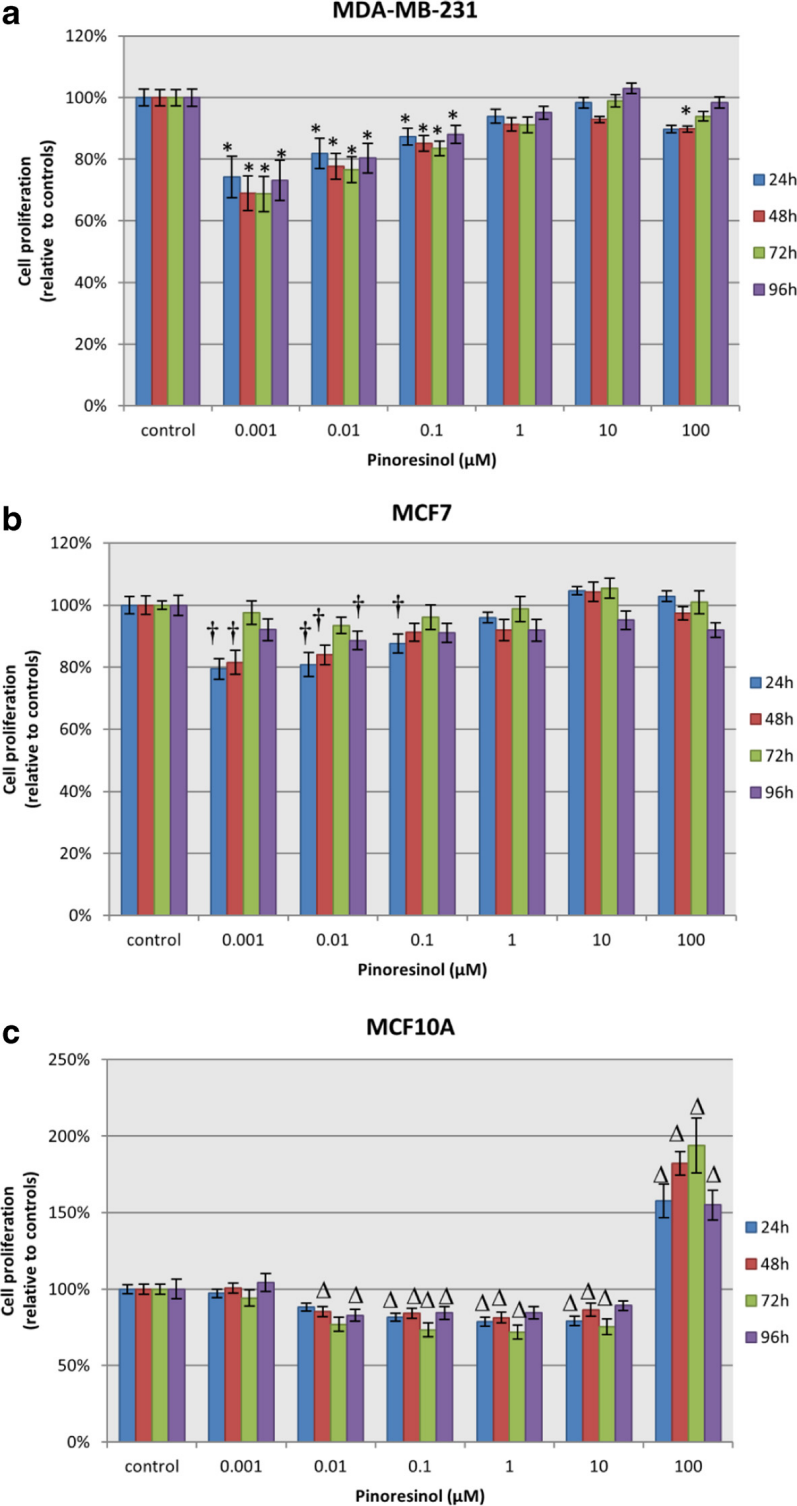
Cell proliferation. Cell proliferation was measured by CellTiter-Blue® after 24 h of (+)-pinoresinol treatment followed by proliferation periods of 24, 48, 72 and 96 h in MDA-MB-231 (**a**), MCF7 (**b**) and MCF10A (**c**) cells. Data are represented as the mean (±SEM) with respect to the controls, which were set as 100 %, for three independent assays carried out in triplicate. *, † and ∆ denote statistically significant differences relative to the control at p < 0.05 for MDA-MB-231, MCF7 or MCF10A, respectively

Proliferation of MDA-MB-231 (Fig. 5a), MCF7 (Fig. 5b) and MCF10A cells (Fig. 5c) was determined after treatment with PINO for 24 h followed by incubation with fresh medium. Measurements were performed at 24, 48, 72 and 96 h following treatment removal. At 0.001, 0.01 and 0.1 μM, cell survival was inhibited in MDA-MB-231 and MCF7 cells. Surprisingly, at 0.001 μM, proliferation was reduced in tumour cells, but not in mammary epithelial cells, in a statistically significant manner. Strong proliferation was observed in MCF10A cells treated with up to 100 μM PINO, whereas neither MDA-MB-231 nor MCF7 showed this effect.

### Analysis of cell cycle and apoptosis

PINO treatment did not produce cell cycle alterations in the three cell lines studied, with the exception of the 100 μM concentration, which diminished the percentage of cells in the S phase in a statistically significant manner (Table 1). This percentage was 8.34 ± 0.94 vs. 17.01 ± 2.33 of the control for MDA-MB-231 (a decrease of 50.97 % respect to the control), 11.96 ± 0.68 vs. 16.72 ± 0.86 for MCF7 (decrease of 28.47 %) and 9.92 ± 1.02 vs. 20.96 ± 1.29 for MCF10A (decrease of 52.67 %). PINO also increased the percentage of cells in G0/G1 for MDA-MB-231 (73.39 ± 1.69 vs. 60.67 ± 4.8 of the control, i.e., increase of 20.97 %) and MCF10A cells (76.1 ± 2.01 vs. 56.45 ± 0.2, that is, 34.81 % of increase respect to the control) and decreased the percentage of cells in 47.24 % in the G2/M phase in MCF10A cells (9.45 ± 2.1 vs. 17.91 ± 1.08 of the control). Unfortunately, 100 μM concentrations are not considered to be physiologically relevant. Representative cell cycle histograms of MDA-MB-231, MCF7 and MCF10A cells treated with PINO are shown in Additional file 1.

**Table 1:** Percentage of cell cycle phases was measured by flow cytometry after treatment with PINO

MDA-MB-231				MCF7				MCF10A				
	SubG0/G1	G0/G1	S	G2/M	SubG0/G1	G0/G1	S	G2/M	SubG0/G1	G0/G1	S	G2/M
Control	1.72 ± 0.3	60.67 ± 4.8	17.01 ± 2.33	20.59 ± 2.46	0.69 ± 0.11	61.57 ± 0.19	16.72 ± 0.86	21.00 ± 0.74	1.23 ± 0.17	56.45 ± 0.2	20.96 ± 1.29	17.91 ± 1.08
0.001 μM	2.57 ± 0.7	60.87 ± 3.37	16.75 ± 1.68	20.23 ± 2.56	0.54 ± 0.03	62.71 ± 0.23	16.70 ± 0.43	20.60 ± 0.73	1.86 ± 0.7	55.32 ± 1.43	22.44 ± 0.34	16.77 ± 1.02
0.01 μM	1.80 ± 0.21	59.16 ± 4.53	17.55 ± 2.68	20.71 ± 2.01	0.62 ± 0.14	61.64 ± 1	16.72 ± 0.25	21.58 ± 0.93	1.67 ± 0.42	54.12 ± 2.3	21.79 ± 0.62	17.36 ± 1.47
0.1 μM	1.32 ± 0.24	59.33 ± 4.15	18.92 ± 1.68	20.35 ± 2.38	0.49 ± 0.02	60.71 ± 0.51	18.41 ± 0.5	20.61 ± 0.64	1.45 ± 0.22	54.84 ± 1.19	20.47 ± 0.53	19.09 ± 1.1
1 μM	1.62 ± 0.29	60.63 ± 4.93	18.56 ± 2.45	19.37 ± 2.48	0.49 ± 0.03	62.99 ± 1.28	16.99 ± 0.62	20.12 ± 1.06	1.85 ± 0.25	54.72 ± 0.65	21.23 ± 0.8	17.58 ± 1.16
10 μM	1.76 ± 0.3	64.85 ± 4.74	17.6 ± 2.38	15.52 ± 2.21	0.57 ± 0.03	61.73 ± 0.65	18.08 ± 0.41	19.62 ± 0.73	1.66 ± 0.12	53.93 ± 1.54	20.23 ± 1.1	19.6 ± 0.84
100 μM	2.29 ± 0.44	73.39 ± 1.69^a^	8.34 ± 0.94^a^	16.06 ± 1.8	0.81 ± 0.19	63.92 ± 1.13	11.96 ± 0.68^a^	23.38 ± 0.85	1.26 ± 0.23	76.1 ± 2.01^a^	9.92 ± 1.02^a^	9.45 ± 2.1^a^

Values represent the percentage of the average ± SEM of three independent experiments. ^a^ indicates statistically significant differences with respect to control (*p* < 0.05)

Statistically significant levels of apoptosis were induced in MCF10A cells treated with 100 μM PINO, with an increment of 445.86 % respect to the control (7.26 ± 2.54 vs. 1.33 ± 0.42) (Table 2). An increase of 19.72 % in apoptosis and 42.98 % in cell death, albeit not statistically significant, also appeared in MDA-MB-231 cells treated at this concentration (18.46 ± 5.92 vs. 14.82 ± 4.76 and 3.42 ± 1.09 vs. 1.95 ± 0.6, respectively). No significant pro-apoptotic effects of PINO were reported in MCF7 cells. Additional file 2 represents the flow cytometry analysis of MDA-MB-231, MCF7 and MCF10A cells after treatment with PINO.

**Table 2:** Percentage of live, apoptotic and dead cells after 24 h after exposure to PINO (0.001, 0.01, 0.1 1 10 and 100 μM)

	MDA-MB-231			MCF7			MCF10A		
	Live	Apoptotic	Death	Live	Apoptotic	Death	Live	Apoptotic	Death
Control	83.22 ± 5.33	14.82 ± 4.76	1.95 ± 0.6	92,03 ± 2,69	6,97 ± 2,9	1,26 ± 0,61	98.14 ± 0.47	1.33 ± 0.42	0.5 ± 0.08
0.001 μM	82.17 ± 4.76	15.56 ± 5.56	2.25 ± 0.94	92,38 ± 1,82	6,2 ± 1,83	1,07 ± 0,62	96.17 ± 0.76	2.73 ± 0.48	1.08 ± 0.29
0.01 μM	80.94 ± 8.15	16.24 ± 6.77	2.8 ± 1.57	93,14 ± 2,06	5,98 ± 1,69	0,85 ± 0,42	95.7 ± 1.44	3.45 ± 1.63	0.83 ± 0.37
0.1 μM	76.84 ± 9.32	20.15 ± 8.06	2.99 ± 1.34	92,47 ± 1,33	6,49 ± 1,08	1,03 ± 0,39	95.11 ± 2.6	3.99 ± 2.56	0.88 ± 0.16
1 μM	78.82 ± 7.41	18.05 ± 6.27	3.16 ± 1.29	93,32 ± 1,98	5,86 ± 1,61	0,8 ± 0,43	93.32 ± 1.51	5.85 ± 1.76	0.8 ± 0.25
10 μM	79.33 ± 7.24	17.75 ± 6.15	2.9 ± 1.19	93,89 ± 1,29	5,4 ± 1,08	0,69 ± 0,28	95.07 ± 1.1	4.17 ± 1.3	0.74 ± 0.2
100 μM	78.1 ± 6.84	18.46 ± 5.92	3.42 ± 1.09	91,65 ± 1,25	7,48 ± 1,14	0,85 ± 0,18	91.82 ± 2.33^a^	7.26 ± 2.54^a^	0.9 ± 0.23

Values represent the average ± SEM of three independent experiments. ^a^ was considered statistically significant respect to the control (*p* < 0.05)

### DCFH-DA

**Fig. 6:**
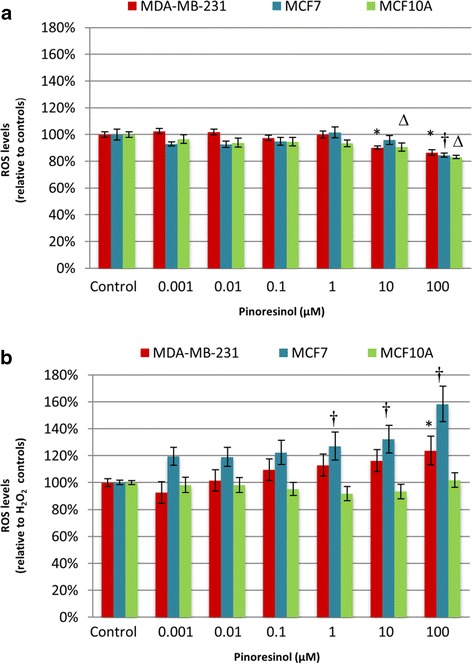
DCFH-DA assay. Intracellular Reactive Oxygen Species (ROS) in breast cells treated with (+)-pinoresinol (in a range from 0.001 – 100 μM) for 24 h under basal conditions **a** and after H_2_O_2_-induced oxidative stress **b**. Data are presented as the mean ± SEM of three independent experiments and *(for MDA-MB-231), † (for MCF7) and ∆ (for MCF10A) represent statistically significant differences (*p* < 0.05) with respect to the control, which was set as 100 %

Reactive Oxygen Species (ROS) were measured using the DCFH-DA method under basal conditions (Fig. 6a) and after oxidative stress induced by H_2_O_2_ (Fig. 6b). In the basal state, 10 and 100 μM concentrations of PINO were shown to diminish ROS levels in a statistically significant way in MCF10A and MDA-MB-231 cells, whereas all concentrations of PINO decreased ROS levels in MCF10A cells (Fig. 6a). Under conditions of oxidative stress (Fig. 6b), the presence of ROS was increased in breast cancer cell lines, especially MCF7 cells, with statistically significant levels observed at 1, 10 and 100 μM in MCF7 cells and at 100 μM in MDA-MB-231 cells. Importantly, increased ROS production was not observed in PINO-treated human mammary epithelial cells (MCF10A).

### Comet assay

**Fig. 7:**
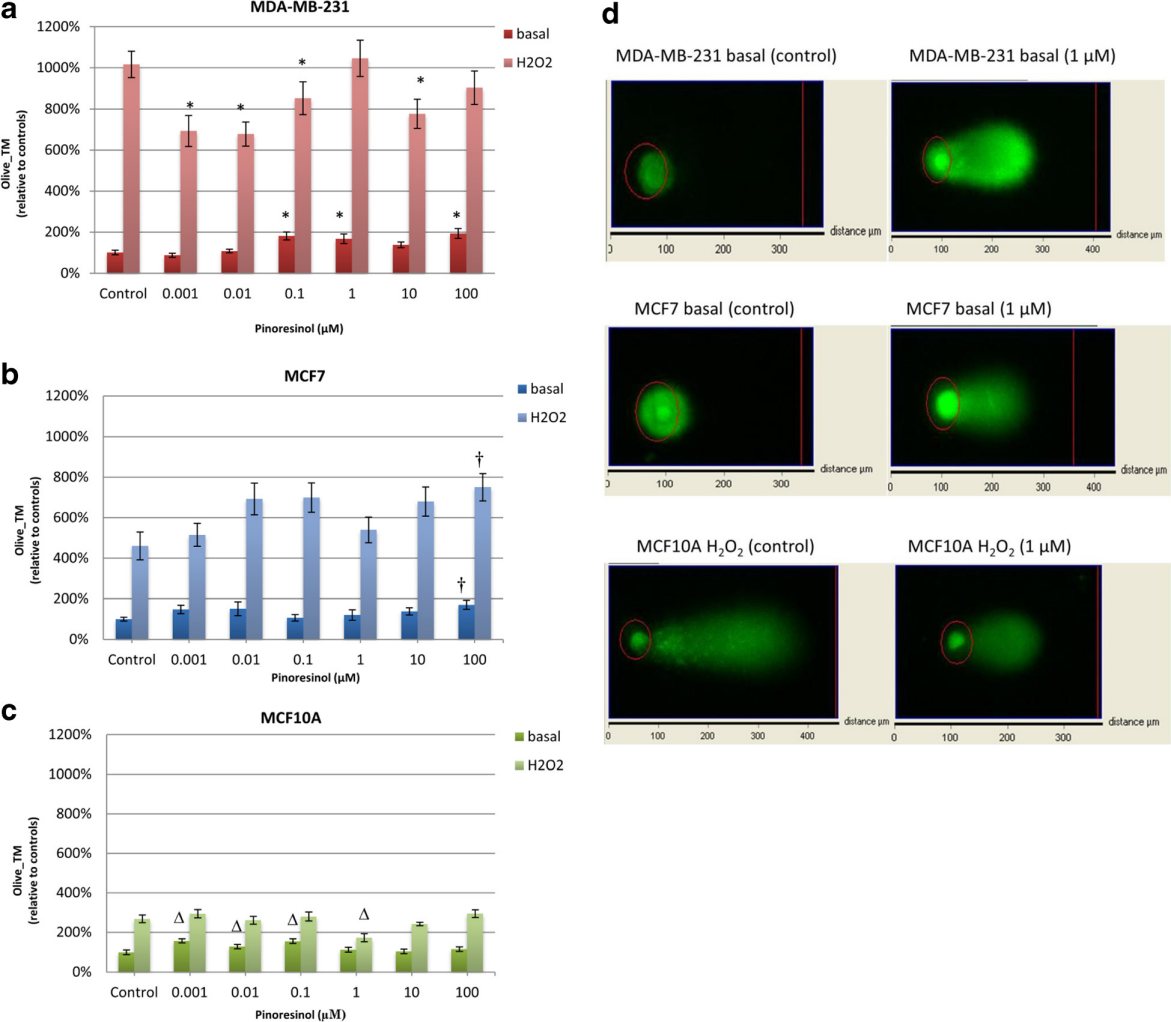
Comet assay. DNA oxidative damage in MDA-MB-231 **a** MCF7 **b** or MCF10A **c** after (+)-pinoresinol treatments expressed as Olive_TM (mean ± SEM). The comet assay was performed under basal and H_2_O_2_-induced injury conditions. Data are expressed as the percentage relative to the basal (untreated) control, which was set as 100 %. Statistically significant differences (*, † or ∆) were established relative to basal or H_2_O_2_-treated control (*p* < 0.05). **d** Representative comet assay images showing different treatments on MDA-MB-231, MCF7 and MCF10A cells

The percentage of DNA damage was determined by alkaline single-cell gel electrophoresis and expressed as Olive_TM. Data were expressed as the percentage relative to the basal (untreated) control, which was set as 100 %. For MDA-MB-231 cells (Fig. 7a), DNA was injured by PINO under basal conditions at 0.1, 1 and 100 μM, but was protected after additional stress with H_2_O_2_. For MCF7 cells (Fig. 7b), treatment with PINO tended to increase DNA damage with respect to both the untreated control and H_2_O_2_-treated control; however, statistically significant changes were only observed at 100 μM. Finally, PINO treatment was shown to have more of a protective effect in MCF10A cells (Fig. 7c) treated with H_2_O_2_ compared to the basal state. Indeed, a statistically significant reduction in DNA damage (93 %) was observed at 1 μM. Fig. 7d shows representative comet assay images of MDA-MB-231, MCF7 and MCF10A cells under different conditions.

## Discussion

As early as 1980s, it was suggested that lignans might prevent breast cancer and that this effect might be correlated with their phytoestrogenic activity. In addition, consumption of VOO, which contains significant amounts of lignans (e.g., PINO and 1-acetoxypinoresinol) as the major components of its phenolic fraction, has been correlated with a low occurrence of breast cancer [1]. In fact, in the phenolic fraction of VOOs there are several compounds with anti-breast cancer properties as oleouropein [33], hydroxytyrosol and tyrosol [6]. Certain compounds showed more effectiveness in ER- breast cancer cells than in ER+ breast cancer cells [33]. PINO and 1-acetoxypinoresinol were first detected in VOO by Owen et al. [8] and differ in their relative amounts according to the different olives varieties used to make the VOO [9]. For example, Brenes et al. [10] reported that Spanish olive oil contains a range of 20 to 45 mg/kg PINO. Despite the well-established preventative role of phytoestrogens against breast cancer, very little research has been done to elucidate whether PINO plays a chemopreventive role or exhibits antitumor activity in human breast cancer cells. Moreover, the oestrogen receptor status is a key factor to consider in breast cancer therapy. In fact, hormone therapy is only used in oestrogen receptor-positive breast cancer [17, 18]. Accordingly, we attempted to elucidate the effects of PINO on human mammary cells with different oestrogen and progesterone receptor expression, to determine whether this compound may contribute, at least in part, to the reduced incidence of breast cancer associated with VOO consumption. For this purpose, we used the following human breast tumour cells: MDA-MB-231 cells (ER-, PR-) and MCF7 cells (ER+, PR+). Furthermore, non-tumorigenic human mammary epithelial cells were also used in the present study [MCF10A (ER-, PR-)].

Our results, summarized in Table 3, indicate that PINO showed cytotoxic, anti-proliferative and pro-oxidant activity in human breast tumour cells, independent of their oestrogen receptor expression levels. In addition, based on its effect in human mammary epithelial cells (Table 3), PINO may have chemopreventive activity, as induced antioxidant activity and prevented DNA from oxidative damage at a concentration of 1 μM. Interestingly, we found that PINO exerted differential activity on human breast tumour cells compared with mammary epithelial cells. Indeed, PINO treatment induced antioxidant activity in mammary epithelial cells, while it acted as a pro-oxidant molecule in human breast cancer cells after inducing oxidative stress.

**Table 3:**
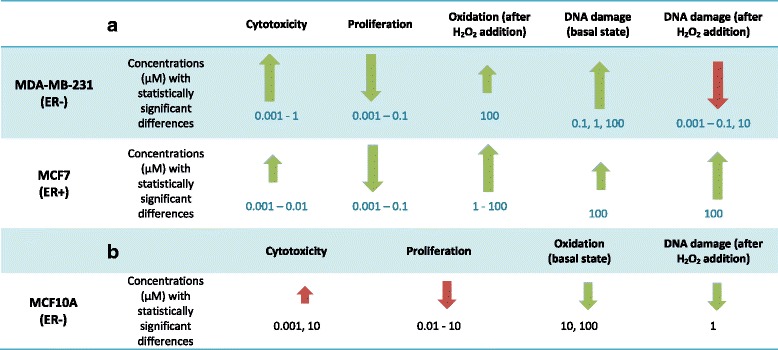
Summarized effects of PINO treatment on tumorigenic and non-tumorigenic mammary cells. a) Human breast tumour cells (MDA-MB-231 and MCF7) and b) non-tumorigenic human mammary epithelial cells (MCF10A)

The cytotoxic activity of PINO on human breast tumour cells is a debated issue. Previously, Chin et al. [25] described that PINO has a cytotoxic effect against MCF7 breast cancer cells (ED_50_ = 4.74 μM); however, in a later article [21], the same author found no cytotoxic effects. Surprisingly, the range of concentrations used in both studies was not specified. In addition, the cytotoxic effects of PINO in MDA-MB-231 cells have not been previously reported. In contrast, we tested a wide range of PINO concentrations and showed that there was cytotoxic activity at different concentrations in both human breast tumour cells tested. While PINO showed cytotoxic activity in both types of human breast tumour cells tested, the effect was more pronounced in negative oestrogen receptor tumour cells compared to oestrogen receptor-positive tumour cells (Figs. 4 and 5). In addition, for the first time, we describe the effects of PINO on human mammary epithelial cells. Our results suggest that PINO ranging between 0.001 and 0.1 μM, which could be considered as physiological doses, has a much greater cytotoxic effect on breast tumour cells compared to mammary epithelial cells, suggesting an anti-tumour effect of this compound with a minor damage to non-tumorigenic tissue.

Little research has been performed to understand the effects of PINO on human breast cancer cell proliferation. Sepporta et al. [27] found that PINO inhibited the growth of MDA-MB-231, but not of MCF7 cells; however, their study was limited to 100 μM, which is not considered to be a physiological concentration. In contrast, we tested a wide range of PINO concentrations, ranging from 0.001 to 100 μM, and showed that low concentrations of PINO elicited a significant antiproliferative effect on both human breast tumour cell lines tested. Future work is needed to clarify the mechanisms of inhibition of breast cancer cells growth only at low doses.

Oestrogen has been associated with the promotion and growth of breast cancer. In line with this result, most human breast cancers that are oestrogen-dependent undergo regression when deprived of the supporting hormone [17]. Our results, therefore, are very interesting because although PINO is a phytoestrogen with an oestrogen-like chemical structure, it produced a decrease in the proliferation of human breast tumour cells. Thus, PINO could have oestrogen antagonist activity, like tamoxifen, which inhibits breast cancer cells proliferation. However, in the experimental cell model we designed, we used cell culture media without oestrogen supplementation, suggesting that PINO is not likely to act as an oestrogen antagonist. Interestingly, a previous prospective study showed that high dietary intakes of plant lignans, such as PINO, were associated with reduced risks of ER+/PR+ postmenopausal breast cancer [20]. We do not believe that the anti-proliferative effects of PINO are mediated by interactions with ERα because this receptor is not expressed in MDA-MB-231 breast tumour cells. Furthermore, the cell proliferation reduction was higher in MDA-MB-231 cells than in ERα + MCF7 breast cancer cells. On the other hand, it is unlikely that the anti-proliferative effects of PINO could be due to the activation of ERβ because both breast cancer cells tested MDA-MB-231 and MCF7, express low levels of this receptor [34]. Additionally, it has been suggested that ERβ exerts anti-proliferative effects in breast cancer cells in the presence of ERα, but exerts proliferative effects in the absence of ERα [17]. If this were true, treatment with PINO would result in an increase of MDA-MB-231 breast cancer cell (ERβ low/ ERα negative) proliferation. Instead, we found an anti-proliferative effect, which was even greater than that observed in MCF7 breast tumour cells (ERβ low/ ERα positive). Based on these results, we hypothesize that the anti-proliferative effects of PINO in the breast cancer cells assayed are independent of both ERα and ERβ status.

Previously, it has been shown that persistent ROS induction in non-tumorigenic cells may lead to cancer initiation, progression and spreading via activation and maintenance of signalling pathways that regulate cellular proliferation, survival, angiogenesis and metastasis [35]. However, we have not found previously published results regarding the antioxidant capacity of PINO in mammary cells. Our results suggest that PINO may prevent cancer development, as it diminished ROS levels in MCF10A mammary epithelial cells.

On the other hand, it is known that cancer cells possess higher intracellular ROS levels than non-tumorigenic cells and that enhanced ROS levels may be exploited to promote cancer cell death [36]. In fact, many of the commonly used chemotherapies are based on increasing oxidative stress above a toxic threshold level to selectively kill cancer cells [36]. In line with this concept, PINO may be used as a potential effective adjuvant to cancer therapies, as it was found to promote ROS generation in breast cancer cells, while it tended to diminish ROS induction in mammary epithelial cells. MCF7 cells were shown to be particularly sensitive to increased ROS levels after H_2_O_2_-induced oxidative stress, which could be related with the levels of DNA damage observed under basal conditions and after oxidative shock. Under basal conditions, PINO also caused DNA damage in MDA-MB-231 cells; however, in contrast, PINO treatment prevented DNA damage in non-tumorigenic mammary epithelial cells, suggesting that PINO treatment may protect DNA in a pro-tumorigenic environment, thereby inhibiting breast cancer initiation and progression. Surprisingly, ER negative cells showed reduced DNA damage in response to H_2_O_2_, whereas ER positive cells showed an increase in DNA damage.

Very few reports have studied the chemical antioxidant capacity of PINO, and the results have varied considerably. For example, Kuo et al. [22] obtained a significant DPPH free radical scavenging activity for PINO, but these results differ from the work done by Chin et al. [21] and Vuorela et al. [23], which demonstrated a much higher IC_50_. Our results suggest that PINO harbours a radical scavenging activity at concentrations of 10 μM or above for ABTS. This capacity was also shown using the DPPH method and is line with work published by Chin et al. [21]. In the ORAC assay, which is considered to be the most biologically relevant assay [24], PINO also showed antioxidant activity in a dose dependent manner.

## Conclusions

Here, we showed that PINO possesses a chemical antioxidant capacity and may have a therapeutic potential to prevent breast cancer development via the reduction of intracellular oxidative stress and DNA damage in human mammary epithelial cells. Furthermore, we showed that PINO promotes an increase in the ROS levels of breast cancer cells after H_2_O_2_ treatment. In sum, this work suggests that PINO may act as adjuvant to pro-oxidative chemotherapies.

Finally, we showed that PINO has anti-tumour effects at low concentrations by promoting cytotoxic, anti-proliferative and pro-oxidant activities in breast cancer cells, independent of their oestrogen receptor status.

## Abbreviations

ABTS, 2,2’-Azino-bis (3-ethylbenzothiazoline-6-sulfonic acid); DCFH-DA, dichlorofluorescin diacetate; DPPH, 2,2-Diphenyl-1-picrylhydrazyl; ER, oestrogen receptor; FBS, foetal bovine serum; HER2, human epidermal growth factor receptor 2; NEAA, non-essential amino acids; ORAC, oxygen radical absorbance capacity; PINO, (+)-pinoresinol; PR, progesterone receptor; ROS, reactive oxygen species; RSA, radical scavenging activity; VOO, virgin olive oil.

## Additional files

Additional file 1: Representative flow cytometry analysis of the cell cycle of MDA-MB-231, MCF7 and MCF10A cells after treatment with (+)-pinoresinol. (DOCX 205 kb) Additional file 2: Representative images of apoptosis analysis by flow cytometry in MDA-MB-231, MCF7 and MCF10A cells. (DOCX 567 kb)

## References

[1] Toledo E, Salas-Salvado J, Donat-Vargas C, Buil-Cosiales P, Estruch R, Ros E (2015). Mediterranean diet and invasive breast cancer risk among women at high cardiovascular risk in the PREDIMED Trial: a randomized clinical trial. JAMA Intern Med.

[2] Sanchez-Quesada C, Lopez-Biedma A, Warleta F, Campos M, Beltran G, Gaforio JJ (2013). Bioactive properties of the main triterpenes found in olives, virgin olive oil, and leaves of Olea europaea. J Agric Food Chem.

[3] Warleta F, Campos M, Allouche Y, Sanchez-Quesada C, Ruiz-Mora J, Beltran G (2010). Squalene protects against oxidative DNA damage in MCF10A human mammary epithelial cells but not in MCF7 and MDA-MB-231 human breast cancer cells. Food Chem Toxicol.

[4] Allouche Y, Warleta F, Campos M, Sanchez-Quesada C, Uceda M, Beltran G (2011). Antioxidant, antiproliferative, and pro-apoptotic capacities of pentacyclic triterpenes found in the skin of olives on MCF-7 human breast cancer cells and their effects on DNA damage. J Agric Food Chem.

[5] Sanchez-Quesada C, Lopez-Biedma A, Gaforio JJ (2015). Maslinic Acid enhances signals for the recruitment of macrophages and their differentiation to m1 state. Evid Based Complement Alternat Med.

[6] Warleta F, Quesada CS, Campos M, Allouche Y, Beltran G, Gaforio JJ (2011). Hydroxytyrosol protects against oxidative DNA damage in human breast cells. Nutrients.

[7] Martin-Pelaez S, Covas MI, Fito M, Kusar A, Pravst I (2013). Health effects of olive oil polyphenols: recent advances and possibilities for the use of health claims. Mol Nutr Food Res.

[8] Owen RW, Mier W, Giacosa A, Hull WE, Spiegelhalder B, Bartsch H (2000). Identification of lignans as major components in the phenolic fraction of olive oil. Clin Chem.

[9] Allouche Y, Jimenez A, Gaforio JJ, Uceda M, Beltran G (2007). How heating affects extra virgin olive oil quality indexes and chemical composition. J Agric Food Chem.

[10] Brenes M, Garcia A, Garcia P, Rios JJ, Garrido A (1999). Phenolic compounds in Spanish olive oils. J Agric Food Chem.

[11] Kulik T, Busko M, Pszczolkowska A, Perkowski J, Okorski A (2014). Plant lignans inhibit growth and trichothecene biosynthesis in Fusarium graminearum. Lett Appl Microbiol.

[12] Yang CP, Huang GJ, Huang HC, Chen YC, Chang CI, Wang SY (2013). The Effect of the Aerial Part of Lindera akoensis on Lipopolysaccharides (LPS)-Induced Nitric Oxide Production in RAW264.7 Cells. Int J Mol Sci.

[13] During A, Debouche C, Raas T, Larondelle Y (2012). Among plant lignans, pinoresinol has the strongest antiinflammatory properties in human intestinal Caco-2 cells. J Nutr.

[14] Wikul A, Damsud T, Kataoka K, Phuwapraisirisan P (2012). (+)-Pinoresinol is a putative hypoglycemic agent in defatted sesame (Sesamum indicum) seeds though inhibiting alpha-glucosidase. Bioorg Med Chem Lett.

[15] Fini L, Hotchkiss E, Fogliano V, Graziani G, Romano M, De Vol EB (2008). Chemopreventive properties of pinoresinol-rich olive oil involve a selective activation of the ATM-p53 cascade in colon cancer cell lines. Carcinogenesis.

[16] Hashim YZ, Rowland IR, McGlynn H, Servili M, Selvaggini R, Taticchi A (2008). Inhibitory effects of olive oil phenolics on invasion in human colon adenocarcinoma cells in vitro. Int J Cancer.

[17] Haldosen LA, Zhao C, Dahlman-Wright K (2014). Estrogen receptor beta in breast cancer. Mol Cell Endocrinol.

[18] Ososki AL, Kennelly EJ (2003). Phytoestrogens: a review of the present state of research. Phytother Res.

[19] Leygue E, Murphy LC (2013). A bi-faceted role of estrogen receptor beta in breast cancer. Endocr Relat Cancer.

[20] Touillaud MS, Thiebaut AC, Fournier A, Niravong M, Boutron-Ruault MC, Clavel-Chapelon F (2007). Dietary lignan intake and postmenopausal breast cancer risk by estrogen and progesterone receptor status. J Natl Cancer Inst.

[21] Chin YW, Chai HB, Keller WJ, Kinghorn AD (2008). Lignans and other constituents of the fruits of Euterpe oleracea (Acai) with antioxidant and cytoprotective activities. J Agric Food Chem.

[22] Kuo PC, Lin MC, Chen GF, Yiu TJ, Tzen JT (2011). Identification of methanol-soluble compounds in sesame and evaluation of antioxidant potential of its lignans. J Agric Food Chem.

[23] Vuorela S, Kreander K, Karonen M, Nieminen R, Hamalainen M, Galkin A (2005). Preclinical evaluation of rapeseed, raspberry, and pine bark phenolics for health related effects. J Agric Food Chem.

[24] Prior RL, Wu X, Schaich K (2005). Standardized methods for the determination of antioxidant capacity and phenolics in foods and dietary supplements. J Agric Food Chem.

[25] Chin YW, Jones WP, Rachman I, Riswan S, Kardono LB, Chai HB (2006). Cytotoxic lignans from the stems of Helicteres hirsuta collected in Indonesia. Phytother Res.

[26] Menendez JA, Vazquez-Martin A, Oliveras-Ferraros C, Garcia-Villalba R, Carrasco-Pancorbo A, Fernandez-Gutierrez A (2008). Analyzing effects of extra-virgin olive oil polyphenols on breast cancer-associated fatty acid synthase protein expression using reverse-phase protein microarrays. Int J Mol Med.

[27] Sepporta MV, Mazza T, Morozzi G, Fabiani R (2013). Pinoresinol inhibits proliferation and induces differentiation on human HL60 leukemia cells. Nutr Cancer.

[28] Re R, Pellegrini N, Proteggente A, Pannala A, Yang M, Rice-Evans C (1999). Antioxidant activity applying an improved ABTS radical cation decolorization assay. Free Radic Biol Med.

[29] Brand-Williams W, Cuvelier ME, Berset C (1995). Use of a free radical method to evaluate antioxidant activity. Lebensm. Wiss. Technol..

[30] Prior RL, Hoang H, Gu L, Wu X, Bacchiocca M, Howard L (2003). Assays for hydrophilic and lipophilic antioxidant capacity (oxygen radical absorbance capacity (ORAC(FL))) of plasma and other biological and food samples. J Agric Food Chem.

[31] Sanchez-Quesada C, Lopez-Biedma A, Gaforio JJ (2015). The differential localization of a methyl group confers a different anti-breast cancer activity to two triterpenes present in olives. Food Funct.

[32] Lee DH, Lim BS, Lee YK, Yang HC (2006). Effects of hydrogen peroxide (H2O2) on alkaline phosphatase activity and matrix mineralization of odontoblast and osteoblast cell lines. Cell Biol Toxicol.

[33] Elamin MH, Daghestani MH, Omer SA, Elobeid MA, Virk P, Al-Olayan EM (2013). Olive oil oleuropein has anti-breast cancer properties with higher efficiency on ER-negative cells. Food Chem Toxicol.

[34] Vladusic EA, Hornby AE, Guerra-Vladusic FK, Lakins J, Lupu R (2000). Expression and regulation of estrogen receptor beta in human breast tumors and cell lines. Oncol Rep.

[35] Glasauer A, Chandel NS (2014). Targeting antioxidants for cancer therapy. Biochem Pharmacol.

[36] Nogueira V, Hay N (2013). Molecular pathways: reactive oxygen species homeostasis in cancer cells and implications for cancer therapy. Clin Cancer Res.

