# Supplementary figures and images for: Phytoestrogen (+)-pinoresinol exerts antitumor activity in breast cancer cells with different oestrogen receptor statuses
Source: BMC Complement Altern Med. 2016 Sep 7;16(1):350. doi: 10.1186/s12906-016-1233-7 (PMC5015324; doi:10.1186/s12906-016-1233-7)

**
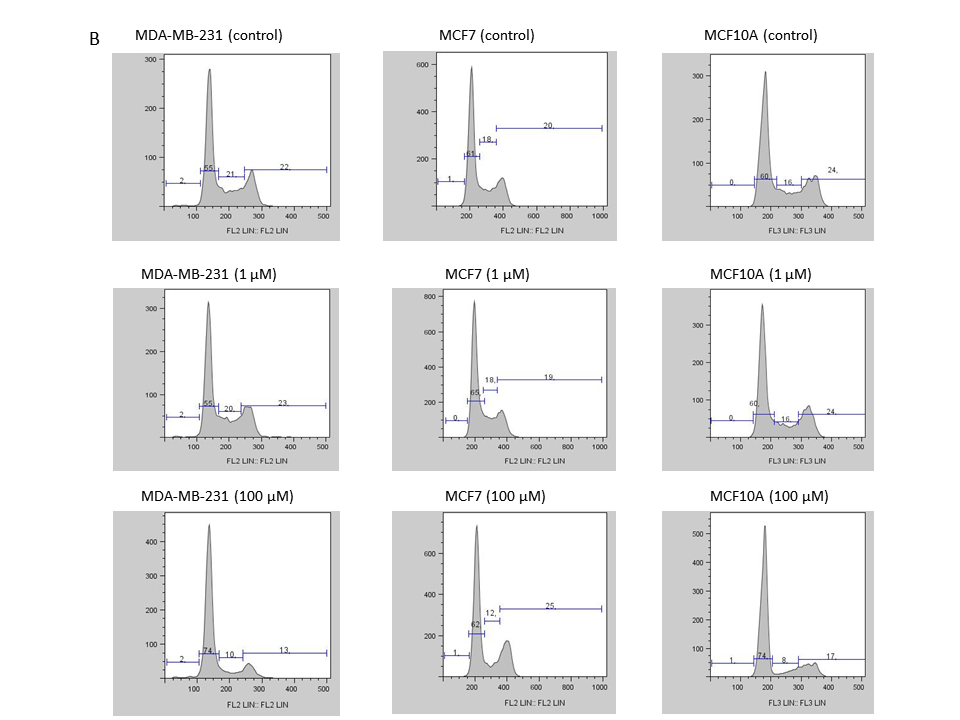
**

Supplement: Additional file 1: — Representative flow cytometry analysis of the cell cycle of MDA-MB-231, MCF7 and MCF10A cells after treatment with (+)-pinoresinol. (DOCX 205 kb) [file 12906_2016_1233_MOESM1_ESM.docx]

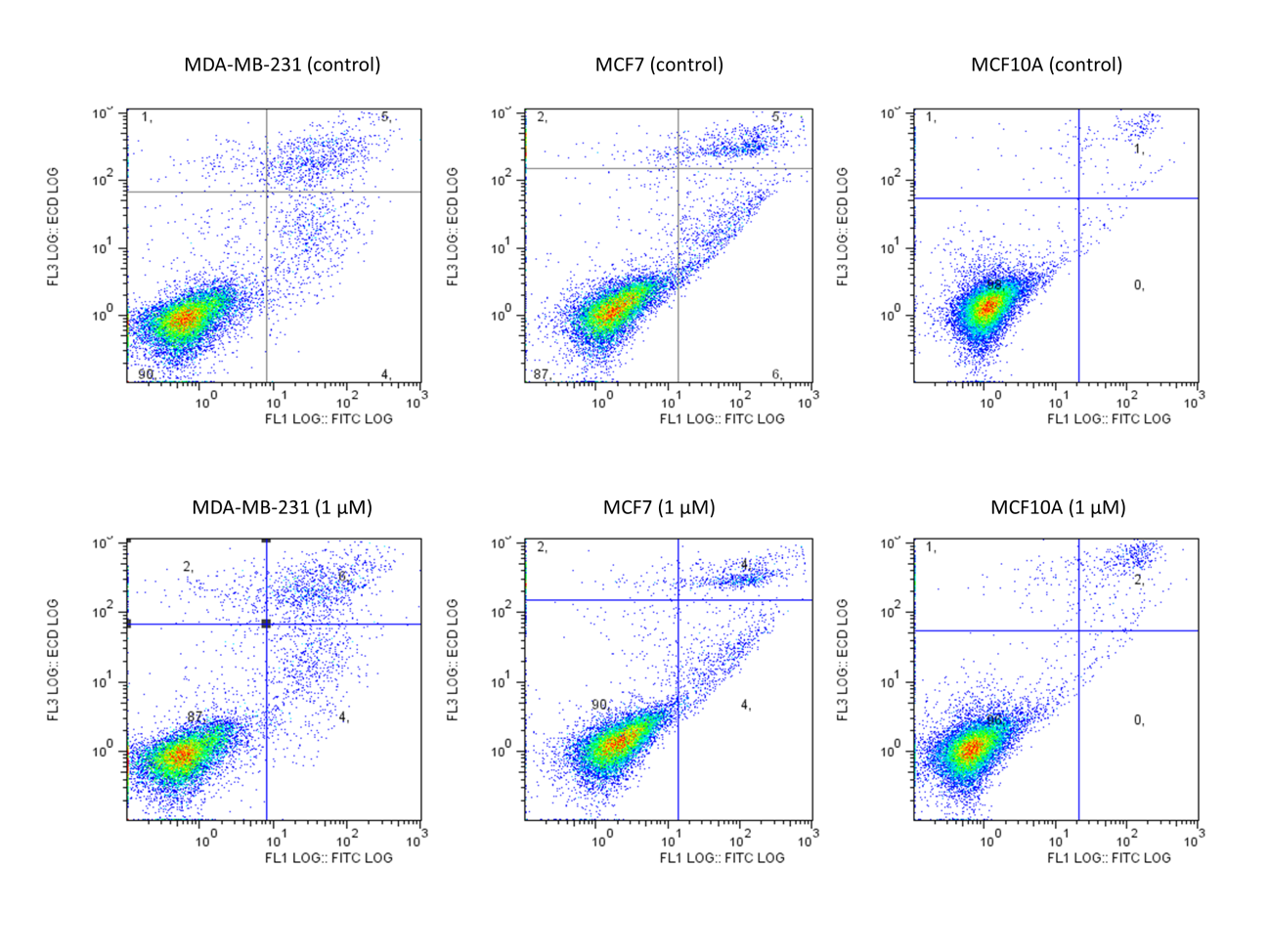


**B**

Supplement: Additional file 2: — Representative images of apoptosis analysis by flow cytometry in MDA-MB-231, MCF7 and MCF10A cells. (DOCX 567 kb) [file 12906_2016_1233_MOESM2_ESM.docx]
